# Multiple Intranasal Foreign Bodies: An Incidental Diagnosis

**DOI:** 10.5005/jp-journals-10005-1078i

**Published:** 2010-09-15

**Authors:** Mohammed Ali Habibullah, Sham S Bhat, Sundeep Hegde K

**Affiliations:** 1 Postgraduate Student, Department of Pedodontics and Preventive Dentistry, Yenepoya Dental College, Derlakatte, Mangalore Karnataka, India; 2 Professor and Head, Department of Pedodontics and Preventive Dentistry, Yenepoya Dental College, Derlakatte, Mangalore Karnataka, India; 3 Professor, Department of Pedodontics and Preventive Dentistry, Yenepoya Dental College, Derlakatte, Mangalore, Karnataka, India

**Keywords:** Intranasal foreign body.

## Abstract

Inquisitive children often insert foreign bodies into their nose or other body orifices while they explore their own bodies in early childhood. Intranasal foreign bodies are found in children, most commonly in 2-4 years olds.

Common symptoms in such cases include pain or discomfort, nasal discharge, nasal congestion, nasal odor, including bromhidrosis (foul body odor). Complications, such as facial cellulitis, epiglottitis, and cephalic tetanus have also been reported. Mentally challenged children may be at a higher risk for such foreign body insertion and may need to be examined at regular intervals.

Careful interpretation of dental radiographs can go a long way in diagnosing such cases especially in the absence of a positive history. Radiolucent objects are more difficult to identify especially in the absence of a positive history, and hence their diagnosis and removal is more challenging for the clinician.

Dental practitioners can play a significant role in the diagnosis of intranasal foreign bodies in children through careful clinical examination and interpretation of dental radiographs.

This case report describes a child referred for dental care and a diagnosis of intranasal foreign body was made based on routine dental panoramic radiograph.

## INTRODUCTION

Inquisitive children often insert foreign bodies into their nose or other body orifices in early childhood. Intranasal foreign bodies are most commonly found in 2-4 years olds^1^ and include toys (beads, marbles), food (peas, seeds, nuts) and others (paper wads, cotton, erasers, pebbles, screws, sponges)^2^ windshield glass,^3^ signet ring,^4^ die^5^ and a 5 cm nail.^6^ Foreign bodies may also be inserted by adults with mental retardation or illnesses, such as schizophrenia.^2^

A majority of the cases are asymptomatic except for a positive history. Symptoms, if present, are commonly pain or discomfort, nasal discharge or congestion. Rare symptoms include bromhidrosis and infections like facial cellulitis and cephalic tetanus.^2^

This case report describes a child referred for dental care and diagnosis of intranasal foreign body made based on routine dental panoramic radiograph.

## CASE REPORT

A 8-year-old male patient reported to our department with a complaint of multiple decayed teeth and swelling in relation to lower left back teeth.

Dental history revealed that the patient had a similar swelling about 2 weeks ago. An OPG was taken and abscess drainage was carried out after antibiotic coverage.

The medical history revealed the patient was a known case of obstructive hydrocephalus and attention deficit hyperactivity disorder (ADHD) and was under medication. He had a history of seizures.

Intraoral examination revealed infected root stumps in relation to 75 with intraoral swelling 1.5 cm wide extending from the gingival margin to the sulcus, soft in consistency and tender on palpation. Root stumps were seen in relation to 54, 64, 74 and 84 deep dentinal caries in relation to 85 and caries in relation to 55 and 83.

Due to the hyperactive nature of the patient, taking radiographs was difficult and it was decided to evaluate the OPG taken 2 weeks ago. The OPG showed a large diffuse radiolucency in relation to 75 approximately 2 cm × 2 cm, extending inferiorly to involve the entire tooth bud of 35, involving both the roots of 75 and mesial root of 36. A provisional diagnosis of radicular cyst was made. Since the radiolucency also involved the crown of 34, a differential diagnosis of dentigerous cyst was suggested.

**Fig. 1:**
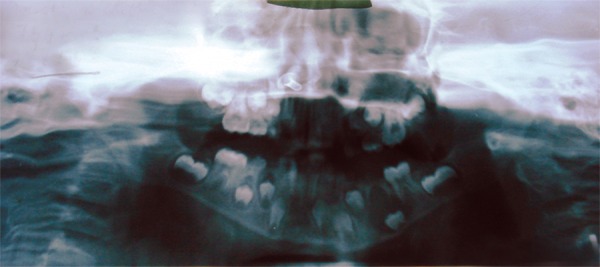
An orthopantanogram of the patient showing the intranasal foreign body

On careful examination of the OPG, a circular radiopacity was observed in the right nasal cavity and a foreign body was suspected (Fig.1). The patient did not give any history of foreign body insertion. On further questioning , the mother gave a history of snoring.

The patient was referred to ENT department. On examination, a button was visualized in the right nasal cavity and successfully removed using a Killian’s nasal speculum. Another gritty object was felt but it could not be removed. The same was scheduled for removal under GA during dental treatment.

Informed written consent was obtained from the parent for dental treatment and foreign body removal under general anesthesia.

Dental treatment undertaken included cyst enucleation in relation to 75, extractions of 54, 64, 74, 36, 84 and 85, restoration of 55 and 83. Pit and fissure sealants were placed in relation to 16, 26 and 36. Histopathological examination confirmed a diagnosis of dentigerous cyst.

**Fig. 2:**
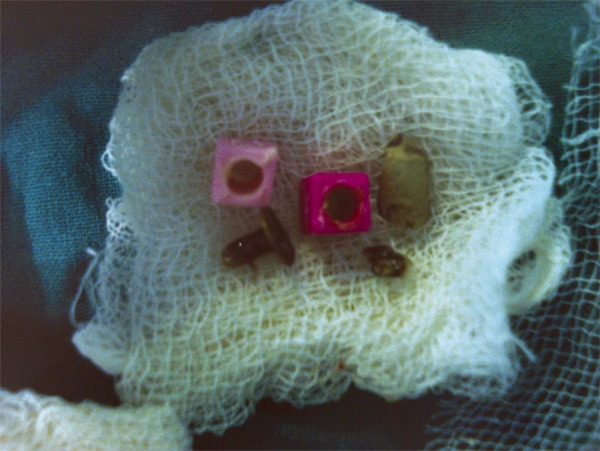
Foreign bodies retrieved under general anesthesia

Foreign body retrieved under GA included 2 beads, 1 tack and piece of eraser from right nostril. A plastic piece was recovered from the left nostril (Fig. 2). Examination of the ear canal was also undertaken as a precautionary measure.

## DISCUSSION

Hydrocephalus is a condition where there is an imbalance between the production and absorption of CSF.^7^ The condition may be congenital or acquired. In this case, the condition was acquired as a result of neonatal meningitis.

Attentiondeficit hyperactivity disorder (ADHD) is one of the most commonly diagnosed mental disorders of children with prevalence ranging from 1.7 to 17%. It is usually diagnosed when children are between 6 and 12 years as they enter the educational system. These children are unable to concentrate on tasks and considered hyperactive. Symptoms include making careless mistakes, being disorganized, having difficulty listening to others and following instructions, restlessness or having trouble being patient and playing quietly.

Some parents and health care providers choose to treat ADHD through behavior modification but the most common treatment is pharmacological therapy.^8^ This child was under medication.

Identification of the nasal foreign body in this patient was significant. Treatment under general anesthesia was scheduled but carried some potential risks especially in the cases of nasal intubation. These include dislodgement with possible aspiration of the foreign body and severe epistaxis.

Radiolucent objects are more difficult to identify, and hence their diagnosis and removal is more challenging for the clinician.^1^ Careful interpretation of dental radiographs can go a long way in diagnosing such cases especially in the absence of a positive history. In this case, the radiopacity of one of the foreign bodies proved to be providential leading to detection and removal of the radiolucent foreign bodies.

The discovery of an occult foreign body in the nasal cavity on routine dental radiography has been previously reported.^1,2^ These cases were diagnosed by using periapical, occlusal and panoramic radiographs. Reports involve objects left *in situ* from 2 to 25 years. The majority of these were asymptomatic. Symptoms when present included nasal obstruction, discharge and facial cellulitis. Most foreign objects discovered from dental radiographs are radiopaque by nature of their inherent properties (e.g, metal tack ) or from the encasing calculus-like material.^2^

The nature of the foreign body will determine the method of removal. Round, slippery objects may be difficult to remove with a grasping instrument but are often easily removed with positive pressure techniques. Soft, friable foreign objects, such as paper or food, may come off piecemeal requiring a combination of picking and suction.^2^

## CONCLUSION

Dental practitioners can play a significant role in the diagnosis of intranasal foreign bodies in children through careful clinical examination and interpretation of dental radiographs. Mentally challenged children may be at a higher risk for such foreign body insertion and may need to be examined at regular intervals. Prompt referral and subsequent removal are essential to avoid complications.
